# Multimodal single-cell omics analysis identifies epithelium–immune cell interactions and immune vulnerability associated with sex differences in COVID-19

**DOI:** 10.1038/s41392-021-00709-x

**Published:** 2021-07-30

**Authors:** Yuan Hou, Yadi Zhou, Michaela U. Gack, Justin D. Lathia, Asha Kallianpur, Reena Mehra, Timothy A. Chan, Jae U. Jung, Lara Jehi, Charis Eng, Feixiong Cheng

**Affiliations:** 1 Genomic Medicine Institute, Lerner Research Institute, Cleveland Clinic, Cleveland, OH USA; 2 Florida Research and Innovation Center, Cleveland Clinic, Port Saint Lucie, FL USA; 3 Department of Cardiovascular and Metabolic Science, Lerner Research Institute, Cleveland Clinic, Cleveland, OH USA; 4 Department of Molecular Medicine, Cleveland Clinic Lerner College of Medicine, Case Western Reserve University, Cleveland, OH USA; 5 Department of Population and Quantitative Health Sciences, Case Western Reserve University, Cleveland, OH USA; 6 Neurological Institute, Cleveland Clinic, Cleveland, OH USA; 7 Center for Immunotherapy and Precision Immuno-Oncology, Cleveland Clinic, Cleveland, OH USA; 8 Department of Cancer Biology, Lerner Research Institute, Cleveland Clinic, Cleveland, OH USA; 9 Department of Genetics and Genome Sciences, Case Western Reserve University School of Medicine, Cleveland, OH USA; 10 Case Comprehensive Cancer Center, Case Western Reserve University School of Medicine, Cleveland, OH USA

**Keywords:** Systems biology, Infectious diseases, Molecular medicine

## Abstract

Sex differences in the susceptibility of SARS-CoV-2 infection and severity have been controversial, and the underlying mechanisms of COVID-19 in a sex-specific manner remain understudied. Here we inspected sex differences in SARS-CoV-2 infection, hospitalization, admission to the intensive care unit (ICU), sera inflammatory biomarker profiling, and single-cell RNA-sequencing (scRNA-seq) profiles across nasal, bronchoalveolar lavage fluid (BALF), and peripheral blood mononuclear cells (PBMCs) from COVID-19 patients with varying degrees of disease severities. Our propensity score-matching observations revealed that male individuals have a 29% elevated likelihood of SARS-CoV-2 positivity, with a hazard ratio (HR) 1.32 (95% confidence interval [CI] 1.18–1.48) for hospitalization and HR 1.51 (95% CI 1.24–1.84) for admission to ICU. Sera from male patients at hospital admission had elevated neutrophil–lymphocyte ratio and elevated expression of inflammatory markers (C-reactive protein and procalcitonin). We found that SARS-CoV-2 entry factors, including *ACE2*, *TMPRSS2*, *FURIN*, and *NRP1*, have elevated expression in nasal squamous cells from male individuals with moderate and severe COVID-19. We observed male-biased transcriptional activation in SARS-CoV-2-infected macrophages from BALF and sputum samples, which offers potential molecular mechanism for sex-biased susceptibility to viral infection. Cell–cell interaction network analysis reveals potential epithelium–immune cell interactions and immune vulnerability underlying male-elevated disease severity and mortality in COVID-19. Mechanistically, monocyte-elevated expression of Toll-like receptor 7 (*TLR7*) and Bruton tyrosine kinase (*BTK*) is associated with severe outcomes in males with COVID-19. In summary, these findings provide basis to decipher immune responses underlying sex differences and designing sex-specific targeted interventions and patient care for COVID-19.

## Introduction

Coronavirus disease 2019 (COVID-19), caused by severe acute respiratory syndrome coronavirus 2 (SARS-COV-2), is a complex disorder with multisystem involvement across different organs.^1–3^ SARS-CoV-2 has infected >31 million people and 565,298 have died in the United States (US) since December, 2019 (as of April 15, 2021).^4^ Approximately 14% of COVID-19-positive patients show severe symptoms associated with advanced age,^1^ sex,^5^ host genetics,^6,7^ disease comorbidities,^1,8^ and other risk factors. Yet, the mechanisms at the cellular and molecular levels underlying these risk factors remain unclear, especially for sex that impacts disease severity.

Sex differences in outcomes have been manifested in multiple infectious diseases, such as influenza,^9^ hepatitis A and C viruses,^10^ and human immunodeficiency virus 1 (HIV1).^11,12^ In addition, HIV1 and hepatitis C virus generally show a higher viral load in men compared to women.^13^ Moreover, women may mount higher immune responses to viral infection and vaccination.^14^ An epidemiologic survey from 1965 through 1997 in the US revealed that 80% of cases across 24 autoimmune diseases occurred in women.^15^ Furthermore, in healthy populations, males have an elevated abundance of CD8^+^ T cells, whereas women have elevated proportion of CD4^+^ T cells and B cells in blood.^16,17^ These studies support a hypothesis that sex differences in immune responses may play crucial roles in the incidence, progression, and outcomes of certain human diseases, including COVID-19.^5^

During the COVID-19 pandemic, men showed a 6.6% increased mortality rate compared to women in the US based on a report of 114,411 COVID-19-related deaths in the National Vital Statistics System from May 1 to August 31, 2020.^18^ Similar observations were also made in United Kingdom, where men had a 1.78 hazard ratio (HR) of COVID-19-related deaths compared to women.^1^ Moreover, male COVID-19 patients have a higher percentage of non-classical monocytes (monocytes-nC) and elevated interleukin (IL)-8 and IL-18 levels in plasma.^5^ Recent animal studies showed elevated viral titer in nasal washings in male hamsters with severe symptoms than in female hamsters.^19^ Owing to heterogeneity of immune cells in the human body, the detailed genetic basis and molecular mechanism of sex differences for sex-specific risk of SARS-CoV-2 infection and disease severity remain unknown. Sex differences in immune responses in COVID-19 may have a direct impact on the efficacy of vaccination and immune-related treatments. Hence, there is a pressing need to better understand the sex-specific heterogeneities of cell subpopulations of the human immune systems and its role in the severity of COVID-19.

In this study, we investigated sex differences in the susceptibility of SARS-CoV-2 infection and severity by combining observations from large-scale patient data from a COVID-19 registry and multimodal single-cell omics analysis of COVID-19 patient samples with varying degrees of disease severity. We identified that male individuals had an elevated susceptibility to severe COVID-19 using propensity score (PS)-adjusted observational analyses. By analysis of laboratory testing data, we found that male individuals had elevated circulating neutrophil–lymphocyte ratio and elevated expression of inflammatory markers compared to females with COVID-19. We further performed multimodal single-cell omics data analysis of nasal tissues and peripheral blood mononuclear cells (PBMCs) isolated from COVID-19 patients to identify differential cell subpopulations contributing to sex differences of human immune responses in COVID-19. In summary, this study provides novel immunological insights into the observed sex-biased susceptibility and disease severity, which may offer precision medicine approaches for the prevention and treatment of male and female individuals with COVID-19.

## Results

### Sex differences of COVID-19 outcomes impacted by age

In total, 27,659 individuals (8361 COVID-19 positive) were tested between March 8 and July 27, 2020 within the Cleveland Clinic Health System in Ohio and Florida, United States (Table 1). We observed that demographic factors (including age and race) have significantly different distributions between men and women in the total cohort of the COVID-19-positive subgroup (Table 1). We found that female (*n* = 16,354) and male (*n* = 11,305) individuals have different percentage of comorbidities relevant to severity of COVID-19,^1,8^ including smoking (*p* < 0.001, two-tailed Fisher’s exact test), diabetes (*p* < 0.001), hypertension (*p* < 0.001), and coronary artery disease (*p* < 0.001). Interestingly, the fraction of COVID-19-positive females (*n* = 4680, 56.0%) was higher than males (*n* = 3681, 44.0%, *p* < 0.001, two-tailed Fisher’s exact test). We found that the sex difference in the occurrence of SARS-CoV-2-positive tests differed by age. For example, the prevalence of COVID-19 positivity was greater in females than that in males only in the age groups >80 years (80–90 years [*p* = 0.006] and >90 years [*p* = 0.029], two-tailed Fisher’s exact test, Supplementary Fig. 1a and Supplementary Table 1). One possible explanation of overall high incidence of SARS-CoV-2 infection in very older female individuals (56.0%) compared to male individuals (44.0%) is that females have longer life span than males.^20^

**Table 1:** Cohort description with the number of patients by sex in a COVID-19 registry

	Total cohort		*p* value	COVID-19 positive		*p* value
	Female	Male		Female	Male	
Patients (*N*)	16,354	11,305		4680	3681	
Age, years (mean (SD))	48.9 (20.6)	50.0 (21.0)	<0.001	49.6 (21.5)	50.6 (19.6)	0.029
White (%)	10,391 (63.5)	7080 (62.6)	0.126	2461 (52.6)	2019 (54.8)	0.042
Black (%)	3822 (23.4)	2489 (22.0)	0.009	1659 (35.4)	1157 (31.4)	<0.001
Race other (%)	828 (5.1)	626 (5.5)	0.087	245 (5.2)	227 (6.2)	0.074
Smoking (%)	1766 (12.3)	1585 (16.6)	<0.001	279 (7.0)	349 (11.7)	<0.001
COPD and emphysema (%)	1217 (10.6)	893 (11.7)	0.016	286 (11.3)	211 (10.8)	0.682
Diabetes (%)	2860 (23.8)	2429 (30.0)	<0.001	771 (28.5)	769 (35.5)	<0.001
Hypertension (%)	6032 (47.3)	4876 (55.8)	<0.001	1787 (57.6)	1548 (62.4)	<0.001
Coronary artery disease (%)	1405 (12.1)	1722 (21.9)	<0.001	364 (14.3)	448 (22.1)	<0.001
Hospitalization (%)	—	—	—	889 (19.0)	957 (26.0)	<0.001
ICU admission (%)	—	—	—	245 (5.2)	365 (9.9)	<0.001
ICU mechanical ventilators (%)	—	—	—	99 (2.1)	174 (4.7)	<0.001

*COPD* chronic obstructive pulmonary disease, *ICU* intensive care unit, *SD* standard deviation

We found that 26% of male patients (*n* = 957, *p* < 0.001, two-tailed Fisher’s exact test, Table 1) vs. 19% (*n* = 889) of females were hospitalized for COVID-19; 9.9% (*n* = 365, *p* < 0.001, two-tailed Fisher’s exact test) of males vs. 5.2% (245) of females were in intensive care units (ICUs); and 4.7% (*n* = 174, *p* < 0.001, two-tailed Fisher’s exact test) of males vs. 2.1% (*n* = 99) of females had to be mechanically ventilated in the ICU. Specifically, the male-biased risks of hospitalization and ICU admission were observed in COVID-19 patients aged from 50 to 90 years (Supplementary Fig. 1a and Supplementary Table 1).

### Sex is linked with elevated susceptibility of SARS-CoV-2 infection and severity

**Fig. 1:**
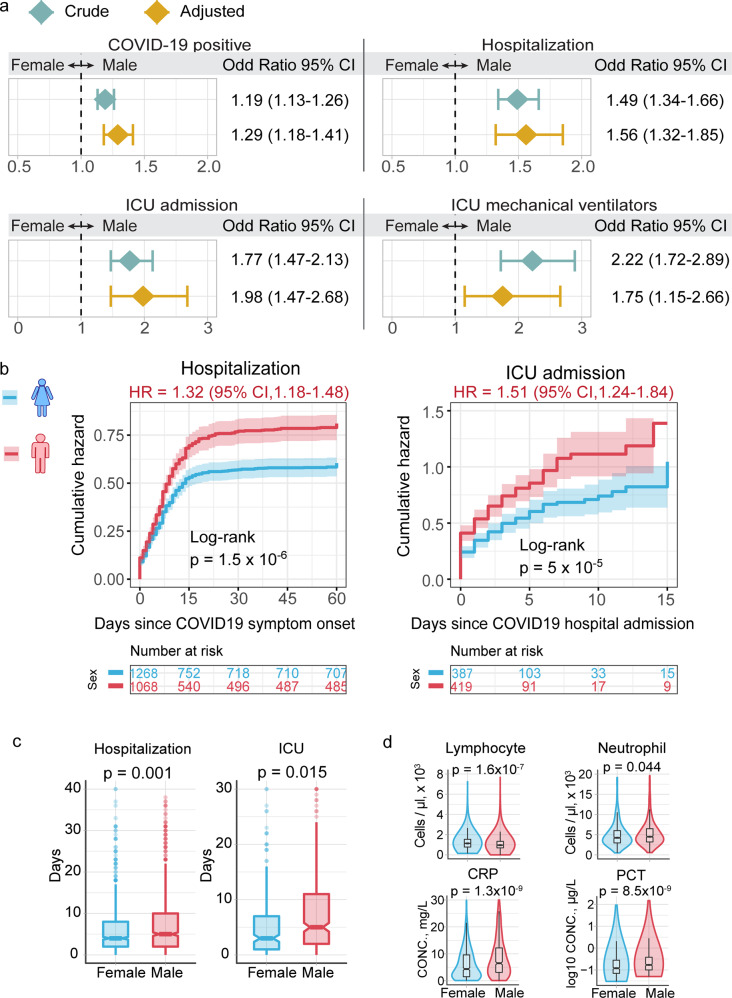
Male individuals are associated with severe COVID-19 outcomes. **a** Odds ratio (OR) analysis between males and females across four COVID-19 outcomes: COVID-19-positive testing by reverse transcription polymerase chain reaction (RT-PCR), hospitalization, intensive care unit (ICU) admission, and usage of ICU mechanical ventilators. Crude cohort means that the OR value was computed based on raw data. PS-adjusted OR analysis: we used propensity score (PS) matching (1:1) population with similar covariate conditions (age, race, smoking, diabetes, hypertension, chronic obstructive pulmonary disease [COPD], emphysema, and coronary artery disease; see “Methods and materials”). See Table 1 for cohort description and sample numbers. **b** Cumulative hazard of hospitalization and ICU admission. All results were computed in the PS-matched groups. Log-rank test with the Benjamini and Hochberg (BH)^51^ adjustment was used to compare the statistical significance of cumulative hazard of hospitalization and ICU admission between males and females. Shadow represents 95% confidence interval (CI). Hazard ratio (HR) was computed using Cox proportional hazards model. **c** Boxplots of the duration of stay in hospital and ICU between male and female individuals. **d** Laboratory testing values for inflammatory markers between male (*n* = 957) and female (*n* = 889) individuals. *p* value was computed by two-sided Wilcoxon rank-sum test

We used adjusted odds ratio (OR) analysis to evaluate the association between sex difference and COVID-19 outcome after adjusting confounding factors using a PS-matching approach. We investigated four types of COVID-19 outcomes: (i) the SARS-CoV-2-positive rate by real-time reverse transcription polymerase chain reaction (RT-PCR), (ii) hospitalization, (iii) ICU admission, and (iv) mechanical ventilation uses in the ICU setting. To reduce risk of confounding factors, we adjusted for age, race, smoking, and four types of disease comorbidities (diabetes, hypertension, chronic obstructive pulmonary disease [COPD], emphysema, and coronary artery disease) based on our sizeable efforts, using the PS-matching method (see “Methods and materials”). We found that male individuals were significantly associated with an increased likelihood of a positive laboratory test result by RT-PCR for SARS-CoV-2 (OR = 1.29, 95% confidence interval [CI] 1.18–1.41, Fig. 1a), COVID-19-related hospitalization (OR = 1.56, 95% CI 1.32–1.85), ICU admission (OR = 1.98, 95% CI 1.47–2.68), and requirement for mechanical ventilation (OR = 1.75, 95% CI 1.15–2.66) after adjusting for confounding factors. These observations together suggest that male individuals have an elevated incidence of SARS-CoV-2 infection and an elevated likelihood of severe COVID-19 outcome compared to females.

To better evaluate sex differences on COVID-19 clinical outcomes, we performed Kaplan–Meier analysis to estimate the cumulative hazard between men and women for admission to hospital and ICU (Fig. 1b and Supplementary Fig. 1b). Men who tested positive for COVID-19 had a higher cumulative hazard for hospitalization than women using both PS-matching (HR = 1.32, 95% CI 1.18–1.48, *p* < 1.5 × 10^−6^, Log-rank test, Fig. 1b) and non-PS-matching methods (HR = 1.43, 95% CI 1.10–1.56, *p* < 1.9 × 10^−14^, Log-rank test, Supplementary Fig. 1b). We found that male patients had a longer duration of hospitalization than female patients (mean [±standard deviation [SD]], 7.6 [±7.8] days vs. 6.2 [±6.7] days, *p* = 0.001, Wilcoxon rank-sum test, Fig. 1c). Specifically, males with COVID-19 have an elevated cumulative hazard for ICU admission compared to females (HR = 1.15, 95% CI 1.24–1.84, *p* < 2.0 × 10^−16^, PS-matching Log-rank test, Fig. 1b). The average duration of ICU stays by male patients was 8.2 (SD = ± 8.9) days, which is significantly longer than 6.2 (SD = ± 7.2) days for female patients (*p* = 0.015, Fig. 1c). Altogether, our analysis suggests that men are significantly associated with severe COVID-19 outcomes compared to women.

### Male-biased COVID-19 severity is associated with elevated inflammation

Hyperinflammation has been reported as a major factor predisposing to a higher mortality in severe COVID-19 patients,^21^ and there is an established sex difference in immune responses.^14^ We next interrogated sex differences in inflammation-related clinical variables available in the COVID-19 registry. We found that the peripheral lymphocyte count was significantly lower in hospitalized male patients (*p* = 1.6 × 10^−7^, Wilcoxon rank-sum test, Fig. 1d) than in hospitalized females. In contrast, the circulating neutrophil levels in hospitalized male patients were higher than that of females (*p* = 0.044, Fig. 1d). In addition, two other inflammatory parameters, C-reactive protein (CRP) and procalcitonin (PCT), were significantly elevated in hospitalized males compared to females, respectively (*p* = 1.3 × 10^−9^ [CRP] and *p* = 8.5 × 10^−9^ [PCT], Fig. 1d). Inflammatory markers, including CRP and PCT, and cell levels of lymphocyte and neutrophil were associated with severity of COVID-19.^22^ Taken together, the elevated levels of CRP and PCT indicated that the male-biased inflammation is potentially associated with poor COVID-19 outcome. In addition, neutrophils have been reported as an essential effector in SARS-CoV-2 infection^23^ and loss of lymphocyte counts has been associated with severity and death of COVID-19 patients.^24^ Altogether, the elevated neutrophil–lymphocyte ratio (Fig. 1d and Supplementary Fig. 1c) implies inflammatory immuno-pathogenesis-mediated severe COVID-19 outcomes in male individuals.^25^ Yet, the underlying mechanisms of male-biased inflammatory responses for COVID-19 patients remain unknown. We therefore turned to investigate the COVID-19 sex difference in human immune systems using multimodal analysis of single-cell RNA-sequencing (scRNA-seq) data.

### Sex-biased cell subpopulations in nasal tissues of critical COVID-19 cases

**Fig. 2:**
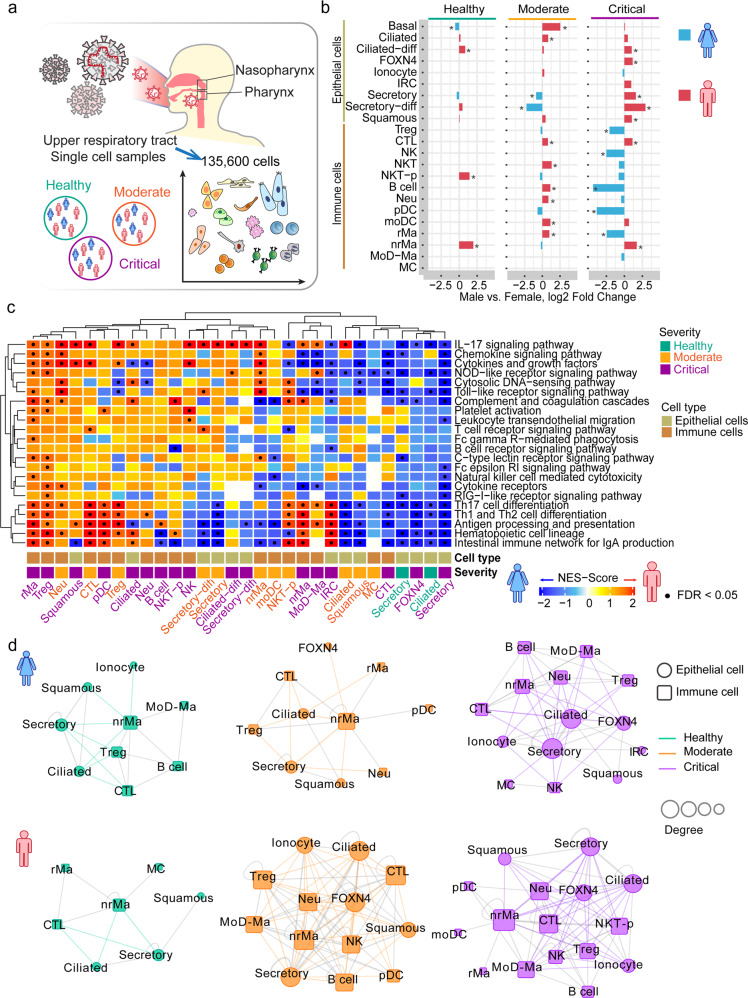
Sex-biased differential cell subpopulation and transcriptional analysis for the upper airway nasal tissues. **a** Sample information of single-cell RNA-sequencing analysis of nasopharynx and pharynx tissues by sex. This dataset includes 135,600 cells from 11 severe COVID-19 patients (3 females and 8 males), 8 mild COVID-19 patients (1 female and 7 males), and 5 healthy controls (3 females and 2 males, Supplementary Table S2). **b** Bar plots showing the log2 fold change of cell subpopulation abundances between male vs. female across healthy donors, moderate, and critical COVID-19 patients. Two-tailed Fisher’s exact test was conducted for each cell type by sex. *p < 0.05. **c** Gene-set enrichment analysis (GSEA) of 22 immune pathways for differentially expressed genes (DEGs) across each cell type of nasal tissues. Cell types having DEGs enriched by at least one significant immune pathway (false discovery rate [FDR] < 0.05) were illustrated in the heatmap. Male-biased genes: the upregulated DEGs in male patients compared to females. Female-biased genes: the downregulated DEGs in male patients compared with females. The gradient color bar shows the normalized enrichment score (NES) scores. Red (NES score > 0) indicates male-biased genes are significantly enriched by immune pathways in a specific cell type. Blue (NES score < 0) indicates female-biased genes are significantly enriched by immune pathways in a specific cell type. Black dots denote FDR < 0.05 (GSEA results are provided in Supplementary Table 4). **d** Sex-biased cell–cell interaction network analyses for nasal single-cell dataset in healthy donors and COVID-19 patients. Significance of ligand–receptor interactions in each cell-type pair were estimated by permutation test with Benjamini–Hochberg-based multiple test correction (FDR < 0.05, see “Methods and materials”). Number of significant interactions in each cell-type pair >30 (top 20%) was used as cutoff to generate the cell–cell interaction network. Circle represents epithelial cell type and square represents immune cell type. The size of nodes denotes the degree (number of connections). Edge colors represent the epithelial–immune cell connections in healthy (green), moderate (orange), and critical (purple) COVID-19 disease condition. Other inter-connections between immune cells or epithelial cells are in gray

We investigated the scRNA-seq profiles^26^ of nasal tissues in the upper airway from SARS-CoV-2-positive patients vs. healthy donors (Fig. 2a). The samples comprised 11 critical COVID-19 patients, 8 moderate COVID-19 patients, and 5 healthy donors (Supplementary Table 2), as described in a previous study.^26^ In total, this scRNA-seq dataset contains 135,600 cells (Fig. 2a) across 22 annotated cell types within two main cell populations (Supplementary Table 3): epithelial cells (9 cell types) and immune cells (13 cell types, Fig. 2b).

From the analysis of relative proportion of each cell type, we observed differential cell types between male and female patients with COVID-19 (Fig. 2b). Compared to the female patients with critical COVID-19, men with critical COVID-19 have elevated abundances across five epithelial cell types: ciliated-differentiating (ciliated-diff; *p* < 2.0 × 10^−16^, Fisher’s exact test), FOXN4-positive cells (FOXN4^+^; *p* = 6.1 × 10^−5^), secretory (*p* < 2.0 × 10^−16^), secretory-differentiating (secretory-diff; *p* < 2.0 × 10^−16^), and squamous cells (*p* < 2.0 × 10^−16^). For immune cells, men with critical COVID-19 have elevated abundances of cytotoxic T cell (CTL; *p* < 2.0 × 10^−16^) and non-resident macrophage (nrMa; *p* < 2.0 × 10^−16^) compared to women (Fig. 2b). In contrast, the abundances of regulatory T cell (Treg; *p* < 2.0 × 10^−16^), natural killer (NK; *p* = 1.2 × 10^−7^), plasmacytoid dendritic cell (pDC; *p* < 2.0 × 10^−16^), and resident macrophage (rMa; *p* < 2.0 × 10^−16^) were decreased in critically ill men compared to critically ill women. These observations suggest differential epithelial and immune cell subpopulations between men and women with COVID-19.

### Male-biased epithelial–immune cell interactions in COVID-19 severity

We next turned to inspect transcriptional activities in sex-biased cell subpopulations from nasal tissues with varying degrees of COVID-19 severity. We performed the gene-set enrichment analysis (GSEA) to evaluate the enrichment of 22 immune pathways at single-cell levels in nasal samples. We defined the differentially upregulated genes in male patients compared with females as male-biased genes based on a previous study.^27^ The differentially downregulated genes in male patients compared with females were defined as female-biased genes. We defined a sex-biased immune cell type in which sex-biased genes are significantly enriched in at least one immune pathway (false discovery rate [FDR] < 0.05, Supplementary Table 4 and Fig. 2c). We found multiple sex-biased immune cells in moderate and critical COVID-19 patients but not in healthy donors. In critical COVID-19 patients, two male-biased rMa and Treg cells have elevated expression in pro-inflammation-related pathways, such as chemokine signaling and cytokines and growth factor pathways (Fig. 2c).

We further inspected cell–cell interactions using the CellPhoneDB algorithm.^28^ First, we quantified the number of significant ligand–receptor interactions (adjusted *p* value <0.05) between cell pairs using scRNA-seq data from nasal tissues (Supplementary Fig. 2b). Meanwhile, we computed three network topological characteristics to measure the cell–cell interaction network (see “Methods and materials,” Supplementary Table 5): (a) number of node, (b) number of edge, and (c) edge-to-node ratio (ENR). We observed that COVID-19 patients have a stronger cell–cell interaction network connectivity (higher number of node and edge) compared to healthy donors (Fig. 2d and Supplementary Table 5). Interestingly, much denser cell–cell interaction networks were observed for both moderate (male ENR = 6.08 vs. female ENR = 2) and critical (male ENR = 4.50 vs. female ENR = 3.57) male COVID-19 patients (Fig. 2d and Supplementary Table 5). In particular, nrMa, Treg, and CTL were highly connected with several epithelial cell types, including squamous, secretory, and ciliated cells in male COVID-19 patients (higher clustering coefficient and degree, Supplementary Table 6). Altogether, these comprehensive network analyses suggest potentially functional roles of epithelial–immune cell interactions underlying male-biased disease severity of COVID-19.

### Male-biased activation of squamous cells upon SARS-CoV-2 infection

**Fig. 3:**
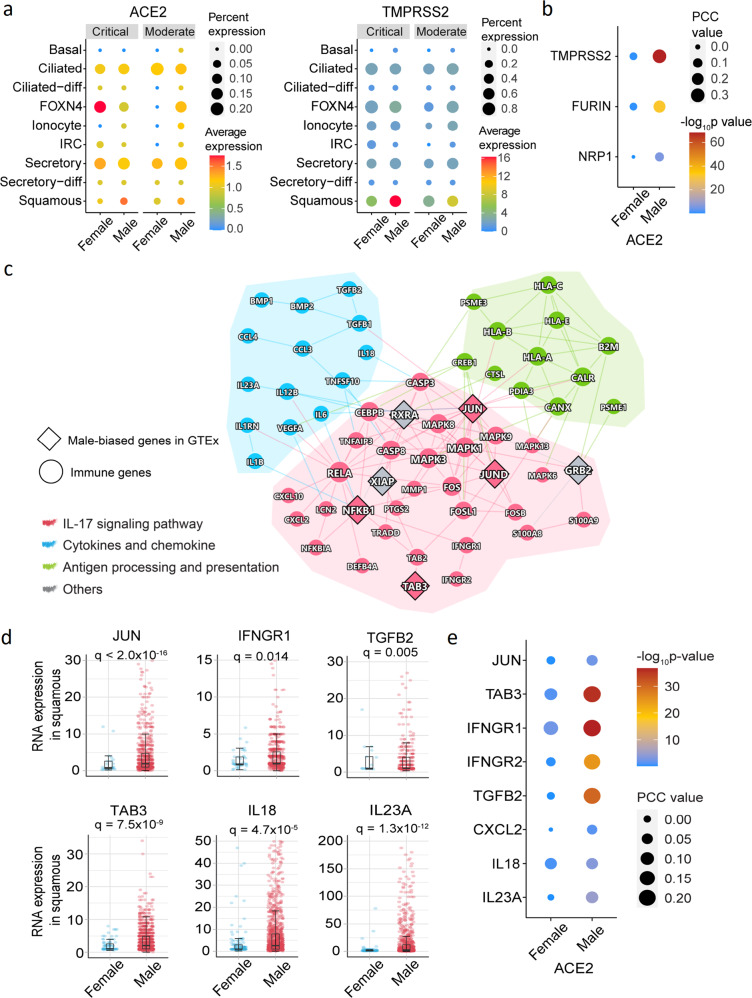
Male-biased transcriptional and network analysis of squamous cells of COVID-19 patients. **a** ACE2 and TMPRSS2 expression in epithelial cell types by sex. Dot size denotes the percentage of cells expressing ACE2 or TMPRSSE. The gradient color bar represents the average expression of genes in each cell type. **b** Co-expression analysis of ACE2 with TMPRSS2, FURIN, and NRP1. Dot size denotes the Pearson Correlation Coefficient (PCC) values. The gradient color bar represents the *p* value (*F*-statistics) of PCC. **c** A highlighted protein–protein interaction subnetwork for male-biased differentially expressed immune genes in squamous cells from the patients with critical COVID-19. The colors for nodes and edges represent different immune pathways. **d** The expression of selected male-biased genes of squamous cells from patients with critical COVID-19. Each dot represents one cell, and the plot only show the cells with positive expression for the genes. Boxplots represent the interquartile range (IQR). Adjusted *p* value (*q*) were computed by the Benjamini–Hochberg method.^51^ **e** Co-expression dot plot of ACE2 with selected immune genes. Dot size denotes the PCC values. The gradient color bar represents the *p* value of PCC

Entry of SARS-CoV-2 into host cells depends on the expression level of the surface receptor angiotensin-converting enzyme 2 (ACE2),^29^ as well as TMPRSS2,^30^ FURIN,^31^ and NRP1.^32^ Yet, the expression levels of ACE2 with either one of the entry factors were unclear at single-cell levels between male and female individuals. We found that the epithelial cells had a higher expression of *ACE2* than immune cells in both men and women (Supplementary Fig. 3a). *ACE2* (adjusted *p* value [*q*] = 0.053, Fig. 3a and Supplementary Table 7), as well as *TMPRSS2* (*q* = 8.1 × 10^−10^) and *FURIN* (*q* = 4.2 × 10^−6^, Supplementary Fig. 3b), have elevated expression in squamous cells from male patients with both moderate and critical COVID-19 compared to females. Figure 3b shows that *ACE2* is significantly co-expressed with *TMPRSS2* (*p* < 2.0 × 10^−16^), *FURIN* (*p* < 2.0 × 10^−16^), and *NRP1* (*p* = 2.3 × 10^−6^) in squamous cells from male patients with critical COVID-19 (Fig. 3b). Yet, *ACE2* is not significantly co-expressed with *TMPRSS2*, *FURIN*, and *NRP1* in females (*p* > 0.05).

We further performed GSEA for sex-biased genes of squamous cells from patients with critical COVID-19. We found that sex-biased genes in squamous cells from critically ill male patients were significantly enriched by 3 immune pathways (Fig. 2c), including the IL-17 signaling pathway (FDR = 0.001), cytokines and growth factors (FDR = 0.001), and antigen processing and presentation (FDR = 0.047). We next turned to identify network modules (defined by the largest connect component [LCC]) for the male-biased immune gene set (upregulated immune genes in men compared to women) of squamous cells. We found that male-biased immune genes formed LCC (*p* = 0.04, permutation test, Fig. 3c) in the human protein–protein interactome network. The proteins in the IL-17 signaling pathway were highly connected to the proteins in the antigen processing and presentation pathway through JUN, which was a male-biased transcription factor reported in Genotype-Tissue Expression (GTEx) database.^33^ TAB3 is an activator of JUN in the IL-17 signaling pathway, and an elevated RNA expression of *TAB3* (*q* = 7.5 × 10^−9^) is observed in squamous cells of male patients with critical COVID-19 (Fig. 3c, d). We found that *TAB3* (also called *MAP3K7IP3*) is an X chromosome-link (X-link) inactivated gene,^33^ and 97% of *TAB3* expression is inactivated by XCI (X chromosome inactivation) in females.^34^ These findings suggest that random X chromosome activation may partially explain sex difference of COVID-19 severity.

Notably, *JUN* and *NFKB1* were enriched among male-biased genes in the GTEx revealed by chromatin immunoprecipitation sequencing in promoter regions.^33^ Specifically, *JUN* and *NFKB1* were highly expressed, with a broader distribution in squamous cells of men compared to women with critical COVID-19 (Fig. 3d and Supplementary Fig. 3c). *JUN* and *NFKB1* were found to induce the expression of multiple pro-inflammatory cytokines/chemokines and their receptors in male squamous cells, including *IFNGR1*, *IFNGR2*, *TGFB2*, *CXCL2*, and *IL18* (Fig. 3c, d and Supplementary Fig. 3c). We also found that the elevated pro-inflammatory cytokines (*IFNGR1*, *IFNGR2*, and *TGFB2*) were significantly co-expressed with *ACE2* in male squamous cells from critically ill COVID-19 patients (Fig. 3e). Altogether, these observations suggest that squamous cells play potential roles in male-biased COVID-19 severity. Further independent cohort validation and functional observations are highly warranted.

### Male-biased immune cell subpopulations in PBMCs from severe COVID-19

**Fig. 4:**
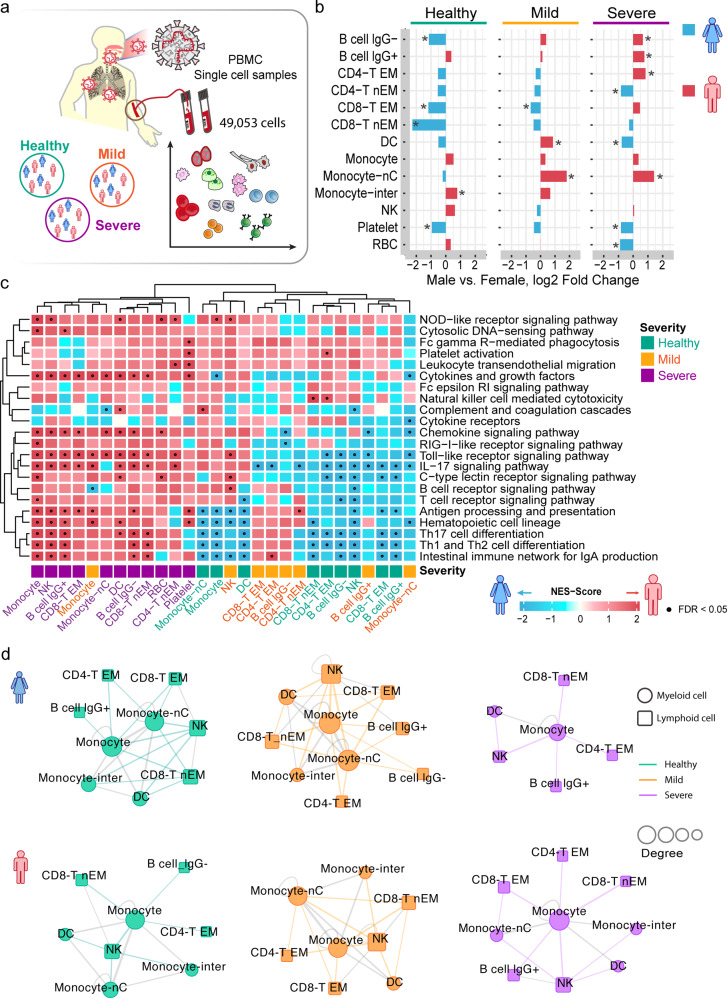
Sex-biased differential cell subpopulation and transcriptional analysis for peripheral blood mononuclear cells (PBMCs). **a** A diagram showing the workflow of single-cell RNA-sequencing analysis of PBMC by sex. This dataset has 49,053 cells from 4 severe patients (2 females and 2 males) and 5 mild patients (3 females and 2 males) and 4 donors in healthy control (3 females and 1 males). **b** Bar plots showing the log2 fold change of male vs. female in cell-type abundances of PBMCs isolated from bloods of healthy donors and patients with mild or severe COVID-19. Two-tailed Fisher’s exact test were conducted for each cell type by sex. *p < 0.05. **c** Gene-set enrichment analysis (GSEA) of 22 immune pathways for DEGs across each cell type of PBMCs. The heatmap illustrates the cell types having DEGs enriched by at least one immune pathway (FDR < 0.05). The details are provided in Fig. 2c and all GSEA results are provided in Supplementary Table 6. **d** Sex-biased cell–cell interaction network analyses for PBMC single-cell RNA-sequencing dataset in healthy donors and COVID-19 patients. Statistical analysis is described in Fig. 2d. Number of significant ligand–receptor interactions in each cell pair .30 (top 20%) was used as cutoff to generate the cell–cell interaction network. Circle represents myeloid cell types and square represent lymphoid cell types. Edge colors represent the myeloid–lymphoid immune cell connections in healthy (green), mild (orange), and severe (purple) COVID-19 disease condition. Other inter-connections between myeloid cells or lymphoid cells are in gray

We further utilized a scRNA-seq dataset from PBMCs of COVID-19 patients (*n* = 9) and healthy donors (*n* = 4, Fig. 4a and Supplementary Table 2) to further inspect immune phenotypes after SARS-CoV-2 infection. In total, we analyzed 49,054 cells and clustered them into 13 annotated cell types based on well-defined marker genes^35^ (Supplementary Fig. 4) and 3 un-annotated cell types (see “Methods and materials”). Compared to females, the male patients with severe and mild COVID-19 had elevated abundances of monocytes-nC (*p* < 2.0 × 10^−16^ [mild] and *p* = 3.7 × 10^−5^ [severe], Fig. 4b). In parallel, we found that male patients with severe COVID-19 have elevated abundances of B cells (lgG− [lgG non-expressed B cell], *p* = 4.7 × 10^−11^ and lgG+ [lgG expressed B cell], *p* = 1.7 × 10^−9^) and CD4-T EM (effector memory-like CD4 T cells, *p* = 1.3 × 10^−9^) but lower abundances of CD4-T nEM cells (non-effector memory-like CD4 T cells, *p* = 1.1 × 10^−7^) and DCs (*p* = 0.016).

We further evaluated immune pathway enrichments across each cell type of PBMCs using GSEA (Supplementary Table 8). We found several sex-biased immune cell types in PBMCs across healthy donors and COVID-19 mild and severe patients as well (Fig. 4c). For example, female-biased monocyte-nC, B cell lgG+, and CD4-T EM cells were observed in mild COVID-19 or healthy donors; yet, male-biased monocytes (including 14 enriched immune pathways [Toll-like receptor (TLR) signaling pathways, RIG-I-like receptor signaling pathway, cytokines and growth factors, and the IL-17 signaling pathway], FDR < 0.05) and NK cells were remarkably observed in severe COVID-19 patients only. These observations suggest elevated pro-inflammatory responses (i.e., monocytes) in male severe COVID-19 patients than in females.

We next inspected the cell–cell interactions of PBMCs using CellPhoneDB.^28^ We found a similar cell–cell interaction network for both female and male patients with mild COVID-19 compared to healthy donors (Fig. 4d and Supplementary 5a). Yet, male patients with severe COVID-19 have elevated pro-inflammatory responses (monocytes, Fig. 4c) and stronger immune cell–cell interactions compared to females (Supplementary Tables 9 and 10), indicating potential immune vulnerability associated with male-biased COVID-19 mortality. For example, the highly immune-activated monocyte (Fig. 4c) interaction with CD8^+^ T cells were predominantly observed in cell–cell interactions of male-derived PBMCs with severe COVID-19 compared to females (Fig. 4d).

### Male-biased activation of monocytes and macrophages in severe COVID-19

**Fig. 5:**
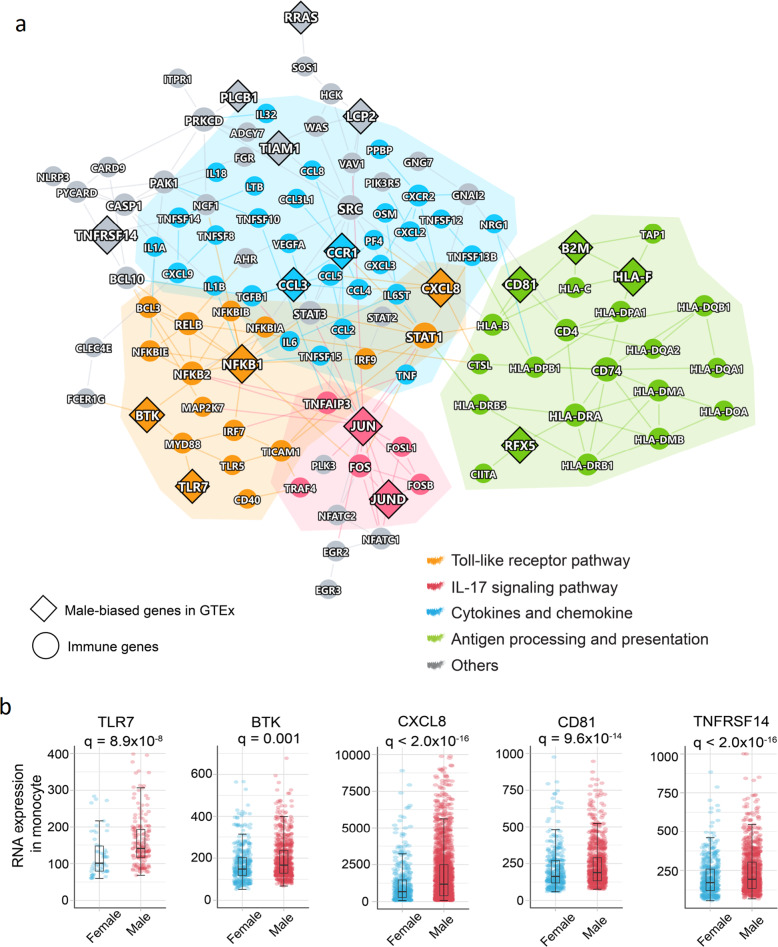
Elevated monocyte immune responses in male patients with severe COVID-19. **a** A highlighted protein–protein interaction subnetwork for male-biased differentially expressed immune genes in monocytes from the patients with severe COVID-19. The colors for nodes and edges represent different immune pathways. **b** Expression of selected male-biased immune genes in monocytes from the patients with severe COVID-19. Each dot denotes one cell, and the plot shows only the cells expressing the genes. Boxplots represent the interquartile range (IQR). *p* value was corrected by Benjamini–Hochberg method^51^

We further performed human protein–protein interactome network analysis to investigate the immunological mechanisms underlying male-biased COVID-19 severity. We found that the male-biased genes in monocytes formed the significant network module (Fig. 5a, *p* < 0.001, Permutation test), which was significantly enriched by several key immune pathways, including TLR pathway (gold), IL-17 signaling pathway (red), cytokines and growth factors (blue), and antigen processing and presentation (green) (Fig. 5a). Several hub genes (including *JUN*, *NFKB1*, *CCR1*, and *SATA1*) are highly connected among different immune pathways. Specifically, *JUN* and *NFKB1* are significantly upregulated in male monocytes with severe COVID-19 compared to females (*q* < 2.0 × 10^−16^).

We next selected genes using subject matter expertise based on a combination of factors: (i) sex-biased expression in GTEx,^33^ (ii) expression quantitative trait locus (eQTL) data availability, (iii) well-annotated immune genes from Kyoto Encyclopedia of Genes and Genomes (KEGG) database,^36^ and (iv) available evidences of X-link genes. Applying these criteria resulted in two top predicted sex-biased genes, including *TLR7* and Bruton tyrosine kinase (*BTK*), which may explain the sex-specific disease severity of COVID-19. Both *TLR7* and *BTK* are X-link inactive genes; 84% of *TLR7* and 98% *BTK* expression are inactivated by XCI in females.^34^ A recent study found a male-biased single-nucleotide polymorphism (SNP) rs2071223 in BTK (*p* = 0.005).^33^ Meanwhile, a rare loss-of-function variant (c.2129_2132del) of *TLR7* was reported in 4 young men with severe COVID-19, which is associated with impaired type I and II interferon responses.^37^ We found that *TLR7* (*q* = 8.9 × 10^−8^) and *BTK* expression (*q* = 0.001) in monocytes are significantly elevated in men with severe COVID-19 compared to women (Fig. 5b). Furthermore, the downstream factors of TLR7 and BTK in TLR pathway, such as *MYD88*, *IRF7*, *NFKB1*, and *JUN*, and cytokines (*TNF*, *IL1B*, and *IL18*) were elevated in men with severe COVID-19 (Fig. 5b and Supplementary Fig. 5b). Altogether, monocyte-specific expression of *TLR7* and *BTK* may provide potential explanations for the male-biased disease severity in COVID-19.

Since the main symptoms of SARS-CoV-2 occurs in human respiratory systems, we further analyzed 3085 SARS-CoV-2-infected positive single cells^38^ of bronchoalveolar lavage fluid (BALF) and sputum samples (*n* = 6 male and n = 2 female severe/critical COVID-19 patients). In total, 8 cell types were investigated, including secretory, squamous, ciliated, macrophage, neutrophil, NK, T cell, and plasma, based on the original study.^38^ Antigen processing and presentation impairs the adaptive immune responses against the virus.^39^ Among the eight cell types, we found that male-biased differentially expressed genes (DEGs) of macrophages (another member of the mononuclear phagocyte system in a component of innate immunity in addition to monocytes) were significantly enriched in the antigen processing and presentation pathway (Supplementary Fig. 6a). In addition, cell–cell interaction analysis using CellPhoneDB^28^ revealed significant ligand–receptor interactions for the squamous–macrophage pair in SARS-CoV-2-positive cells isolated from BALF and sputum of male patients (Supplementary Fig. 6b). Recent studies showed that macrophages contribute to SARS-CoV-2 transmission^40,41^ and elevated inflammation.^42^ In summary, these observations show that macrophages may play important roles in sex differences of SARS-CoV-2 pathogenesis.

## Discussion

In this study, we investigated the sex differences in disease severity and mortality between male and female individuals, using large-scale COVID-19 patient registry, multimodal analysis of scRNA-seq profiles, and cell–cell interaction network analysis. We identified that male patients with COVID-19 had a higher rate of hospitalization and ICU admission and a longer stay time in hospital or ICU compared to female individuals (Fig. 1), which is consistent with an observational study using 17 million COVID-19 tested populations.^1^ Via analysis of laboratory testing data in the COVID-19 patient registry database, we found that serum of male individuals has elevated inflammatory markers (CRP and PCT) compared to females with COVID-19, suggesting sex-specific inflammatory responses underlying sex differences between male and female individuals with COVID-19. We also tested T cell and B cell profiles of lymphocytes using a flow cytometry dataset^43^ from patients with mild (16 males vs. 11 females) and severe COVID-19 (22 males vs. 18 females). We found that there is no significant difference of T cells and B cells between female and male patients with mild or severe COVID-19 (Supplementary Fig. 8), consistent with a recent study.^5^

We further performed multimodal analysis of scRNA-seq data from nasal tissues, PBMCs, BALF, and sputum with varying degrees of COVID-19 pathology. We identified that sex-biased, differential immune cell types and gene transcriptional networks provide potential molecular mechanisms for the male-biased susceptibility of SARS-CoV-2 infection and severity. For example, male-biased genes identified in squamous and nrMa cells are significantly enriched in the cytokines and growth factors and IL-17 signaling pathway (Figs. 2c and 3), revealing the elevated pro-inflammation in male patients with severe COVID-19. Compared to female patients, we observed that male patients with moderate or severe COVID-19 showed differentil abundances of NKT, B cells, and Neu. For example, a longitudinal study revealed that the individuals with moderate COVID-19 had productive innate (Neu) and adaptive immune (B cell) responses (NKT involving in both innate and adaptive immunity); yet, severe COVID-19 patients showed dysregulated immune responses.^44^ As shown in Fig. 2b, male-decreased abundances of Neu and NKT (productive innate immunity) in critical COVD-19 compared to moderate patients revealed that the loss of innate immunity may be associated with COVID-19 severity. Male-decreased B cells in critical COVD-19 compared to moderate patients suggested that impairment of the adaptive immune responses against the virus may be associated with COVID-19 severity as well.

In this study, we found elevated immune responses in both male and female individuals; yet, we observed more elevated pro-inflammatory responses in males (Fig. 4c). In agreement with recent studies,^21,45^ hyper-inflammation (induced by pro-inflammatory cytokines and chemokines) is a major factor predisposing to high mortality in severe COVID-19 patients. We found more elevated pro-inflammatory responses in male PBMC samples with severe COVID-19 (Figs. 4 and 5). For example, pro-inflammatory cytokines and chemokines CXCL8 (*q* < 2.0 × 10^−16^, Fig. 5b), IL18 (*q* = 0.009), and IL1B (*q* < 2.0 × 10^−16^, Supplementary Fig. 5b) were elevated in monocytes from male patients with severe COVID-19 compared to females. The predisposition to a pro-inflammatory state is a major contributor to immune vulnerability.^46^ In summary, these observations suggest that immune vulnerability may be associated with male-biased morbidity and mortality in severe COVID-19, such as elevated monocyte-related pro-inflammatory responses.

TMPRSS2, an S-protein priming protease, facilitates viral entry into the human upper respiratory tract. Its high expression is a predictor of an enhanced efficiency of SARS-CoV-2 infection and greater severity of COVID-19.^30^ Aligned with the observation in nasal samples, *TMPRSS2* (*q* = 9.8 × 10^−7^) showed significant upregulation in SARS-CoV-2-positive squamous cells isolated from male BALF and sputum comparing to females (Supplementary Fig. 7a); yet, *TMPRSS2* expression did not correlate with SARS-CoV-2 viral load (viral reads per million, Supplementary Fig. 7b). In addition, other SARS-CoV-2 entry factors, such as *ACE2*, *FURIN*, and *NRP1*, do not show differential expression in SARS-CoV-2-positive squamous cells between males and females (Supplementary Fig. 7a). There are several possible explanations. For example, SARS-CoV-2 may be cleared by human after disease progression during viral load measure. In addition, there may be unknown, sex-specific host factors of SARS-CoV-2, in addition to *ACE2*, *FURIN*, and *NRP1*.

TLR7 and BTK are male-biased genes in peripheral monocytes with severe COVID-19. TLR7 escapes the XCI in female B cell, resulting in the higher expression levels in women than in men.^47^ Young men with severe COVID-19 were found to carry the four nucleotide deletion in TLR7 (c.2129_2132del; pGln710Argfs*18), while the affected family members carried only one missense variant on TLR7 (c.2383G>T; pVal795Phe).^37^ This evidence supports the potential role of TLR7 in the male-biased mortality in severe COVID-19 patients (Fig. 5b). BTK, a tyrosine kinase, was identified as a top male-biased gene in monocytes of severe COVID-19 patients. BTK is an X-linked gene and 98% of its expression is inactivated by XCI^34^ in females. By analyzing gene expression profiles of 838 subjects from the GETx,^33^ we found an eQTL SNP (rs2071223) on BTK in male-derived lymphocytes (*p* = 0.005), further supporting the male-specific role of BTK in COVID-19. Several BTK inhibitors (e.g., acalabrutinib and ibruitinib, which blocks TLR7-dependent NF-κB activation in monocytes) have been shown to be potentially promising in treatment of patients with severe COVID-19. Altogether, these observations emphasize that sex is a key biological variable in predicting the efficacy of pharmacologic treatments (such as BTK inhibitors) in people diagnosed with COVID-19.

We acknowledge several potential limitations. Due to the sex-biased mortality in COVID-19, small sample size of female patients from scRNA-seq datasets used in this study may influence the findings. Thus, the sex-biased cell types and gene transcriptional networks we identified should be validated further in a large cohort of both male and female individuals. We observed that the male patients aged between 30 and 80 years have a greater risk of SARS-CoV-2 infection (Supplementary Fig. 1a); yet, older female individuals aged ≥80 years have a higher incidence of SARS-CoV-2 infection. Exploring sex differences and underlying immune mechanisms in younger COVID-19 patients, including the pediatric population, may provide more actionable biomarkers and immune targets for disease prevention and vaccine development.^48,49^ Yet, the underlying molecular mechanisms and genetic factors for COVID-19 sex differences are warranted further studies and clinical validations. For example, genetic basis of sex differences should be investigated in the future using the genetic datasets from the growing, diverse COVID-19 population, such as the genome-wide association studies from COVID-19 Host Genetics Initiative.^50^

Taken together, our analysis provides a comprehensive understanding of the clinical characteristics and immunological mechanisms underlying sex differences in COVID-19. We found that the male-biased genes identified in squamous (including *TAB3*, *TGFB2*, and *IL18*) from nasal samples and in monocyte cells (*TLR7*, *BTK*, and *CXCL8*) from PBMC samples are significantly enriched in the cytokines and growth factors and TLR pathway (Figs. 2c, 3, 4c, and 5), revealing that male-elevated pro-inflammation was associated with disease severity of COVID-19. Mechanistically, epithelium–immune cell interactions and immune vulnerability from cell–cell interaction network analysis further support inflammation-associated disease severity and mortality in male COVID-19 patients. In particular, monocyte-elevated expression of two key inflammation-associated genes, *TLR7* and *BTK*, is associated with severe outcomes in males with COVID-19. If broadly applied, these findings will offer a path toward sex-specific molecularly targeted prevention and therapeutic development for COVID-19, which will be essential against the COVID-19 pandemic and future pandemics from other emerging pathogens in a sex-specific manner.

## Methods and materials

### COVID-19 registry

We used an institutional review board–approved COVID-19 registry dataset, including 27,659 individuals (8274 positive) tested during March to July 2020 from the Cleveland Clinic Health System in Florida and Ohio. All tested samples were pooled nasopharyngeal and oropharyngeal swab specimens. SARS-CoV-2 infection was confirmed by RT-PCR in the Cleveland Clinic Robert J. Tomsich Pathology and Laboratory Medicine Institute. All SARS-CoV-2 testing was authorized by the Food and Drug Administration under an Emergency Use Authorization and accord with the guidelines established by the Centers for Disease Control and Prevention.

The COVID-19 registry includes COVID-19 test results, baseline demographic information, medications, and disease conditions and others. We conducted a series of retrospective studies to test the sex difference with four COVID-19 outcomes. Data were extracted from electronic health records (EPIC Systems) and were manually checked by a study team trained on uniform sources for the study variables. We collected and managed all patient data using REDCap electronic data capturing tools. Statistical analyses for smoking, diabetes, hypertension, COPD, emphysema, and coronary artery disease were conducted after adjusting missing values.

### PS-matching analysis

We select case–control PS method to match the four COVID-19 outcomes: (i) positive COVID-19 test: COVID-19-positive patients were matched to negative patients (*n* = 19,298) in total COVID-19 testing patients; (ii) Hospitalization: hospitalized patients (*n* = 1846) were matched to non-hospitalized patients (*n* = 6515) in COVID-19-positive patients; (iii) ICU admission: the patients of ICU admission (*n* = 610) were matched to non-ICU admission patients (*n* = 1236) hospitalized due to COVID-19; (iv) ICU mechanical ventilator use: the COVID-19 patients used mechanical ventilator (*n* = 273) were matched to non-mechanical ventilator user (*n* = 337) in ICU. To reduce the bias from confounding factors, all PS-matched patients were adjusted for age, race, smoking, and presence of diabetes, hypertension, COPD, emphysema, and coronary artery disease. PS matching was conducted with matchit package in the R v3.6.3 platform.

### Clinical outcome analysis

OR was used to measure the association between the COVID-19 outcomes and sex based on logistic regression model. An OR > 1 means that male is associated with a higher likelihood of the outcome, and an OR < 1 indicates lower likelihood of the outcome for male. Kaplan–Meier method was used to estimate cumulative hazard of hospitalization and ICU admission of COVID-19-positive patients by sex. And Cox proportional regression model was used to quantify the hazard of sex for COVID-19 outcomes. For hospitalization outcome, time to event was defined as the duration from the start date of COVID-19 symptom onset to hospital admission date. For ICU admission outcome, time to event was defined as the duration from the date of hospital admission to the date of ICU admission. Log-rank test was used for comparison among different sex groups and adjusted using the Benjamini and Hochberg (BH) method.^51^ All the cumulative hazard analyses were performed using the Survival and Survminer packages in R v3.6.3.

### Single-cell RNA-seq analysis

In this study, we used two single-cell datasets of COVID-19 patients vs. healthy control (Supplementary Table 2) and one SARS-CoV-2-positive cell dataset.

Dataset-1^26^ (European Genome-phenome Archive repository: EGAS00001004481). Nasopharyngeal and pharyngeal tissues were collected from COVID-19-positive patients (11 severe patients with 3:8 female vs. male ratio, and 8 mild patients with 1:7 female vs. male ratio) and healthy controls (5 healthy donors with 3:2 female vs. male ratio). The dataset contains 135,600 cells with cell type annotated. Twenty-two cell types were annotated based on the marker gene expression (Supplementary Table 3) in the original study.^26^ The basal (*FABP5*, *SERPINB3*, *TMSB4X*), secretory (*XBP1*), ciliated cells (*EFHC1*, *CCDC153*), and their differentiating cell types, including ciliated-diff (*EFHC1, CCDC153*, *MLF1*) and secretory-diff (*XBP1*, *VMO1*), are major epithelial cell types of the airways. FOXN4^+^ cell is a transient cell state of multi-ciliated differentiation^52^ and ionocytes (*FOXI1*, *CFTR*) differentiate from basal.^53^ Both of them are typical cell types tracing the dynamics of epithelial differentiation on SRAS-CoV-2 infection. The squamous cells were characterized by a strong expression of *SPRR3* and *SPRR2A*.

Immune cell types rMa (*CD74*, *HLA-DRA*, Supplementary Table 3), nrMa (*IL1B*, *VCAN*, *CD14*), moMa (MD macrophage, *CD14*, *CXCL10*, *IFIT1*), neutrophil (*CD16*, *LYN*, *FCGR3B*), pDC (*CD137*, *IRF7*, *IL3RA*), and moDC (MD dendritic cell, *CD74*, *HLA-DRA*) are myeloid cell classes (*CD11b*^*+*^). T cells (cytotoxic T cells labeled by *CD4*, *GZMA*, and *GZMB*, and T regular labeled by *CD3G* and *KLRB1*), B cells (*CD19*), NK cells (*NCAM1*, *FCGR3A*, and *KLRD1*), and plasma cells (*CD27*, *SDC1*, and *CD79A*) are lymphoid cell types.

Dataset-2^35^ (GSE149689) was downloaded from the NCBI GEO database. This dataset included three groups, patients infected with influenza A, patients infected with COVID-19, and healthy controls. We only focused on the COVID-19 and healthy control populations in this study. For the COVID-19 group, PBMC samples were collected from 4 severe patients (1:1 female vs. male ratio) and 5 mild patients (3:2 female vs. male ratio). In addition, the healthy control group has 4 donors in with 3:1 ratio in female vs. male. Qualifying cells based on the criteria from the original paper were used for the single-cell analysis. In total, 49,053 cells were used to downstream analysis. We used the cell-type markers from a previous study^35^ (CD3E, CD4, CCR7, CD8A, NCAM1, CD14, FCGR3A, NR4A1, CD19, FCER1A, PPBP, and HBB, Supplementary Fig. 4b).

Dataset-3^38^ is a scRNA-seq dataset from BALF and sputum along with available viral load data. In total, we analyzed 3085 SARS-CoV-2-infected positive cells in BALF and sputum samples (male *n* = 6 vs. female *n* = 2) from severe/critical COVID-19 patients. We used eight known cell types, including secretory, squamous, ciliated, macrophage, neutrophil, NK, T cell, and plasma, based on the original study.^38^

All single-cell analyses and visualizations were performed with the R package Seurat v3.1.4.^54^ “NormalizeData” was used to normalize the data. “FindIntegrationAnchors” and “IntegrateData” functions were used to integrate cells from different samples. tSNE was used as the dimensionality reduction method for visualization. “FindAllMarkers” function with the MAST test as the finding marker method for each cell type. edgeR^55^ v 3.12 was used to find the DEGs (log fold change [log_2_FC] > 0.5 and FDR < 0.05) for each cell type between males vs. females.

### Cell–cell interaction analysis

Cell–cell interaction analysis was performed by CellPhoneDB^28^ (v2.1.4) (https://github.com/Teichlab/cellphonedb) on python 3.7 platform. Permutation test repeated 1000 times was used to evaluate the significance for ligand–receptor pairs across each cell type. All *p* values were further corrected by the BH method.^51^ We also computed several network topological characteristics^56^ for the cell–cell interaction networks, including number of nodes, number of edges, ENR, clustering coefficient, degree, and average shortest path length (Supplementary Tables 5, 6, 9, and 10) using the NetworkX package (https://networkx.github.io/) on Python 3.7 platform.

### Immune gene set enrichment analysis

To evaluate the immune pathway activity in females and males, GSEA was conducted as described in the previous work.^57^ The immune gene profiles were retrieved from KEGG database.^36^ We selected 22 immune-related pathways and 1241 genes from KEGG belonging to the immune system subtype. For each cell type, we performed a GSEA on the list of DEGs ranked by the log_2_FC. The normalized enrichment score (NES; Eq. 1) was calculated for 22 immune pathways in male- and female-specific gene sets (Supplementary Fig. 2a):

1 $${{{\mathrm{NES}}}} = \frac{{{\rm{ES}}}}{{\overline {{\rm{ES}}_{{{{\mathrm{permutation}}}}}} }}$$

where ES^57^ denotes enrichment score. Normalization of the enrichment score reduced the effect of the differences in gene set size and in correlations between gene sets and the expression dataset. We defined the upregulated DEGs in male vs. females as male-biased genes based on a previous study.^27^ The downregulated DEGs in male vs. females were defined as female-biased genes. NES score >0 and FDR < 0.05 indicates that male-biased genes in a specific cell type are significantly enriched by immune pathways, while NES score <0 and FDR < 0.05 indicates that female-biased genes in a specific cell type are significantly enriched by immune pathways. Permutation test (1000 times) was performed to evaluate the significance. All analyses were performed with the prerank function in GSEApy package (https://gseapy.readthedocs.io/en/master/index.html) on Python 3.7 platform.

### Building the human protein–protein interactome

To build a comprehensive human interactome, we assembled in total 18 bioinformatics databases to collect protein–protein interactions (PPIs) with five types of experimental evidences: (1) literature-curated PPIs identified by affinity purification followed by mass spectrometry (AP-MS) and literature-derived low-throughput experiments from BioGRID,^58^IntAct,^59^ Instruct,^60^ MINT,^61^ PINA v2.0,^62^ and InnateDB^63^; (2) binary PPIs tested by high-throughput yeast-two-hybrid (Y2H) systems from two public available high-quality Y2H datasets^64–66^; (3) kinase–substrate interactions by literature-derived low- or high-throughput experiments from Kinome NetworkX,^67^ Human Protein Resource Database (HPRD),^68^ PhosphositePlus,^69^ PhosphoNetworks,^70^ Phospho.ELM,^71^ and DbPTM 3.0^72^; (4) signaling network by literature-derived low-throughput experiments from SignaLink 2.0^73^; and (5) protein complex data identified by a robust AP-MS methodology collected from BioPlex v2.0.^74^ The final human protein–protein interactome used in this study included 351,444 unique PPIs (edges or links) connecting 17,706 proteins (nodes). The detailed description for building human protein–protein interactome are provided in our recent studies.^66,75^

### Identification of cell-type-specific and sex-biased immune gene networks

We picked the overlap genes between sex-specific differential gene sets and 1241 immune genes (22 immune pathways from KEGG^36^) as sex-biased immune gene set for each cell type. In addition, we identified immune genes as highly confident sex-biased genes based on the following criteria: (1) the genes that were X-chromosome-linked genes from GTEx^33^ and literature evidence; (2) sex-biased transcription factors and other genes in specific tissues or cell types from GTEx; and (3) the genes that were significantly associated with sex-biased eQTL in specific tissues or cell types (the solid tissues in which majority cell types are epithelial cell, blood, and lymphocytes). Thereafter, we picked the largest connected component from sex-biased immune gene set based on PPIs as final sex-biased immune gene module in a specific cell type. This step was performed with the NetworkX package (https://networkx.github.io/) on Python 3.7 platform.

### Statistical analysis and network visualization

Fisher’s exact tests for categorical data were performed by SciPy 1.2.1 (https://www.scipy.org/). One-way analysis of variance was used to compare the difference of continuous clinical variable by sex. *p* < 0.05 were considered significant. Networks were visualized using Cytoscape.

## Supplementary information

Supplementary Materials

Supplementary Table 1

Supplementary Table 2

Supplementary Table 3

Supplementary Table 4

Supplementary Table 5

Supplementary Table 6

Supplementary Table 7

Supplementary Table 8

Supplementary Table 9

Supplementary Table 10

## Data Availability

All codes and data used in this study are freely available: https://github.com/ChengF-Lab/COVID-19Sex. Other data are available in Supplementary file and other codes used in this study are available upon reasonable correspondence to the corresponding authors. The final .rds files are available at https://figshare.com/s/1e9dc06d2b80b7361f99.
