# Supplementary material for: Multimodal single-cell omics analysis identifies epithelium–immune cell interactions and immune vulnerability associated with sex differences in COVID-19
Source: Signal Transduct Target Ther. 2021 Jul 30;6:292. doi: 10.1038/s41392-021-00709-x (PMC8322111; doi:10.1038/s41392-021-00709-x)
Supplement: Supplementary file 1 — Supplementary Materials [file 41392_2021_709_MOESM1_ESM.docx]

Supplementary Materials for

**Multimodal Single-Cell Omics Analysis Identifies Epithelium-Immune Cell Interactions and Immune Vulnerability Associated with Sex Differences in COVID-19**

Yuan Hou, Yadi Zhou, Michaela U. Gack, Justin D. Lathia, Asha Kallianpur, Reena Mehra, Timothy Chan, Jae U. Jung, Lara Jehi, Charis Eng, Feixiong Cheng

Correspondence to: Feixiong Cheng ([chengf@ccf.org](mailto:chengf@ccf.org))

**This PDF file includes:**

Figures. S1 to S8

**Other Supplementary Materials for this manuscript include the following:**

Table S1 (Excel document)

Table S2 (Excel document)

Table S3 (Excel document)

Table S4 (Excel document)

Table S5 (Excel document)

Table S6 (Excel document)

Table S7 (Excel document)

Table S8 (Excel document)

Table S9 (Excel document)

Table S10 (Excel document)


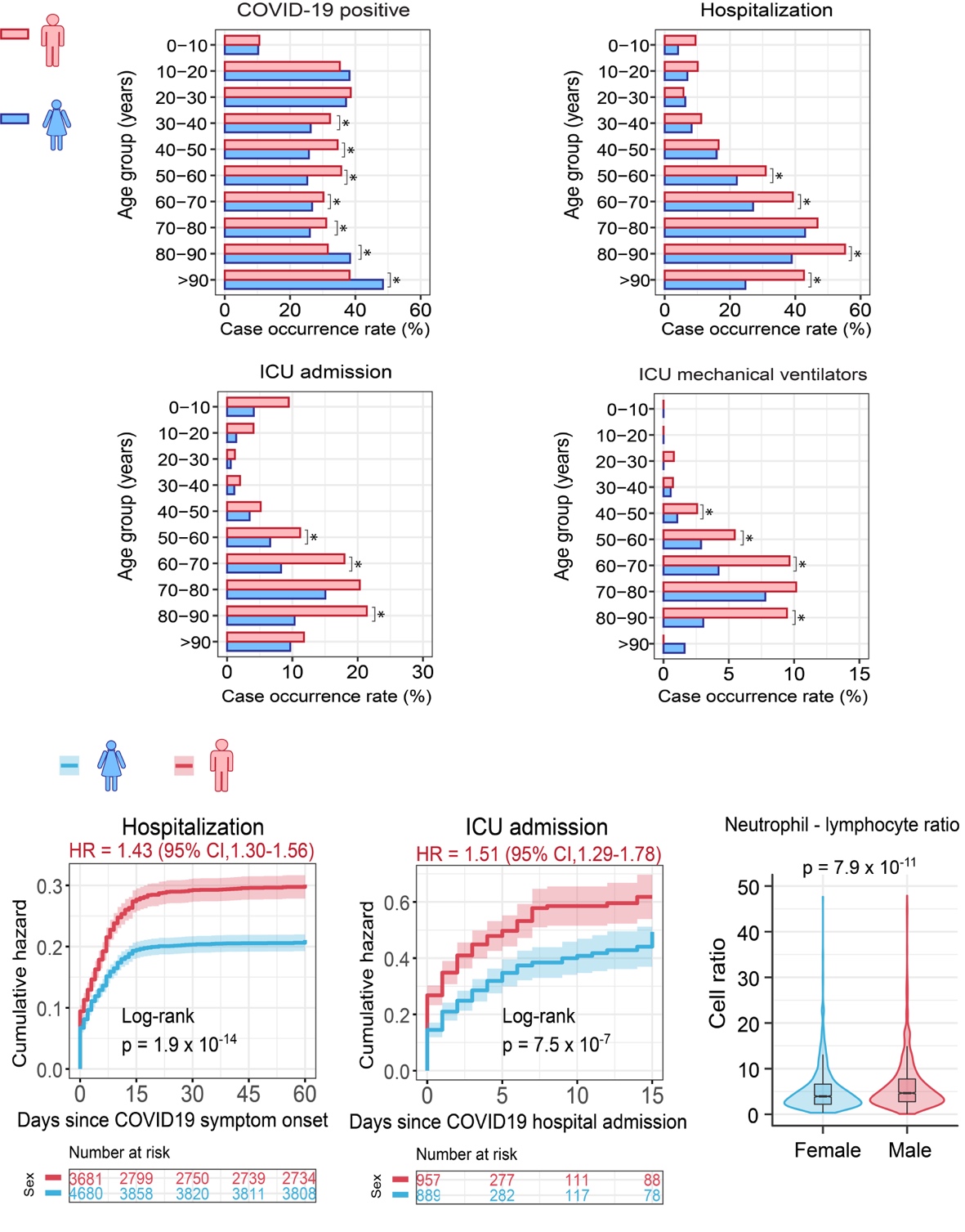


**Supplementary Fig. 1** **Clinical outcome and characteristics between male and female individuals with COVID-19.** **a** Statistics analysis of four COVID-19 outcomes across different age groups. * denote p < 0.05 using two-tailed Fisher’s extract test. **b** Cumulative hazard of hospitalization and ICU admission are shown. The log-rank test with the BH adjustment was used for comparing the statistical significance of cumulative hazard of hospitalization and ICU admission between men and women. The shadow represents 95% confidence interval. HR, hazard ratio. **c** Neutrophil to lymphocyte ratio between male (n = 957) and female (n = 889) individuals. P-value was computed by two-sided Wilcoxon rank-sum test.


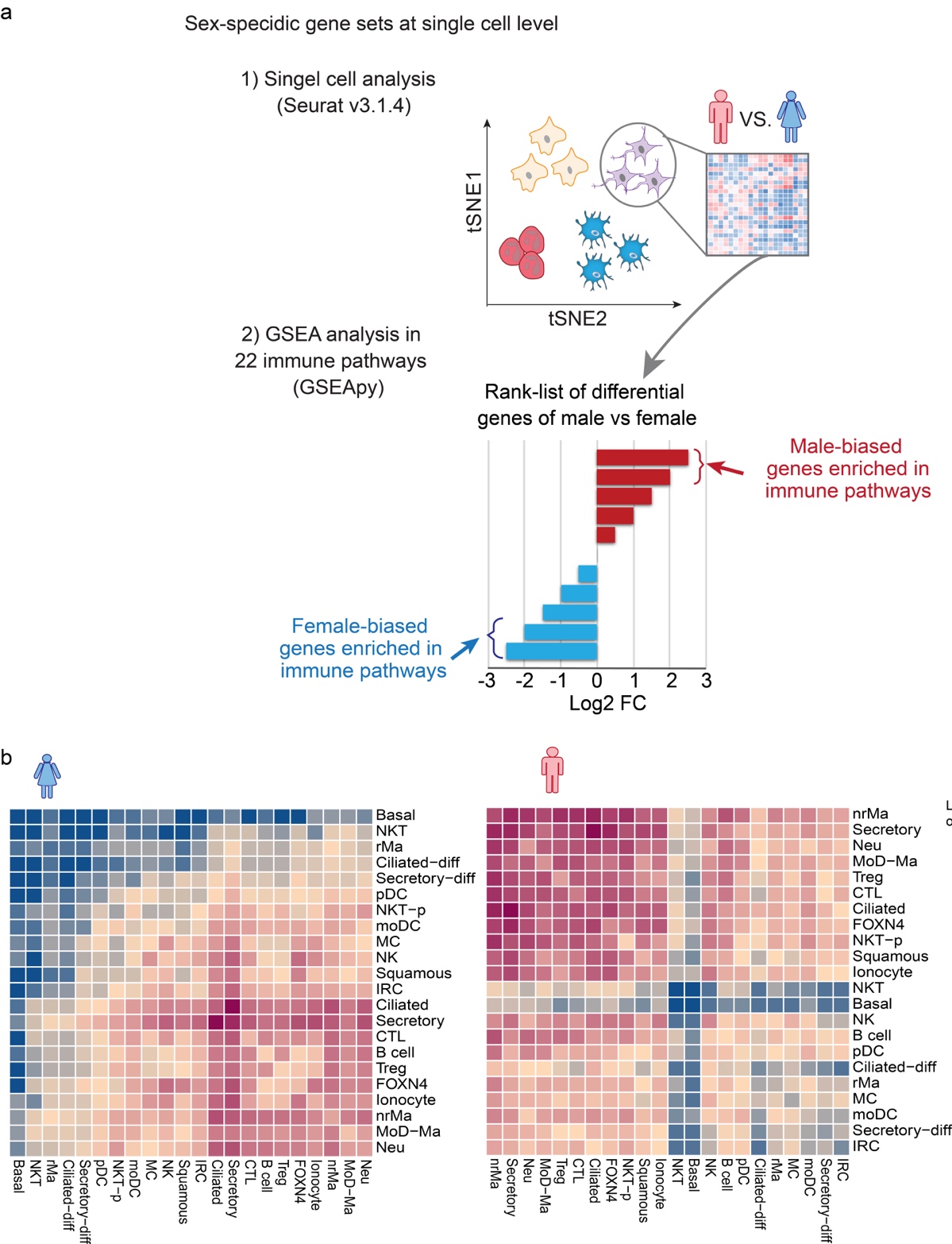


**Supplementary Fig. 2** **Cell types analysis of nasal samples by sex**. **a** Workflow of GSEA analysis. We defined the differentially up-regulated genes in male patients compared with females as male-biased genes. Otherwise, the top differentially down-regulated genes in male patients compared with females as female-biased genes. **b** Heatmap showed the number of significant ligand-receptor interactions between cell pairs in male and female patients with critical COVID-19.

**
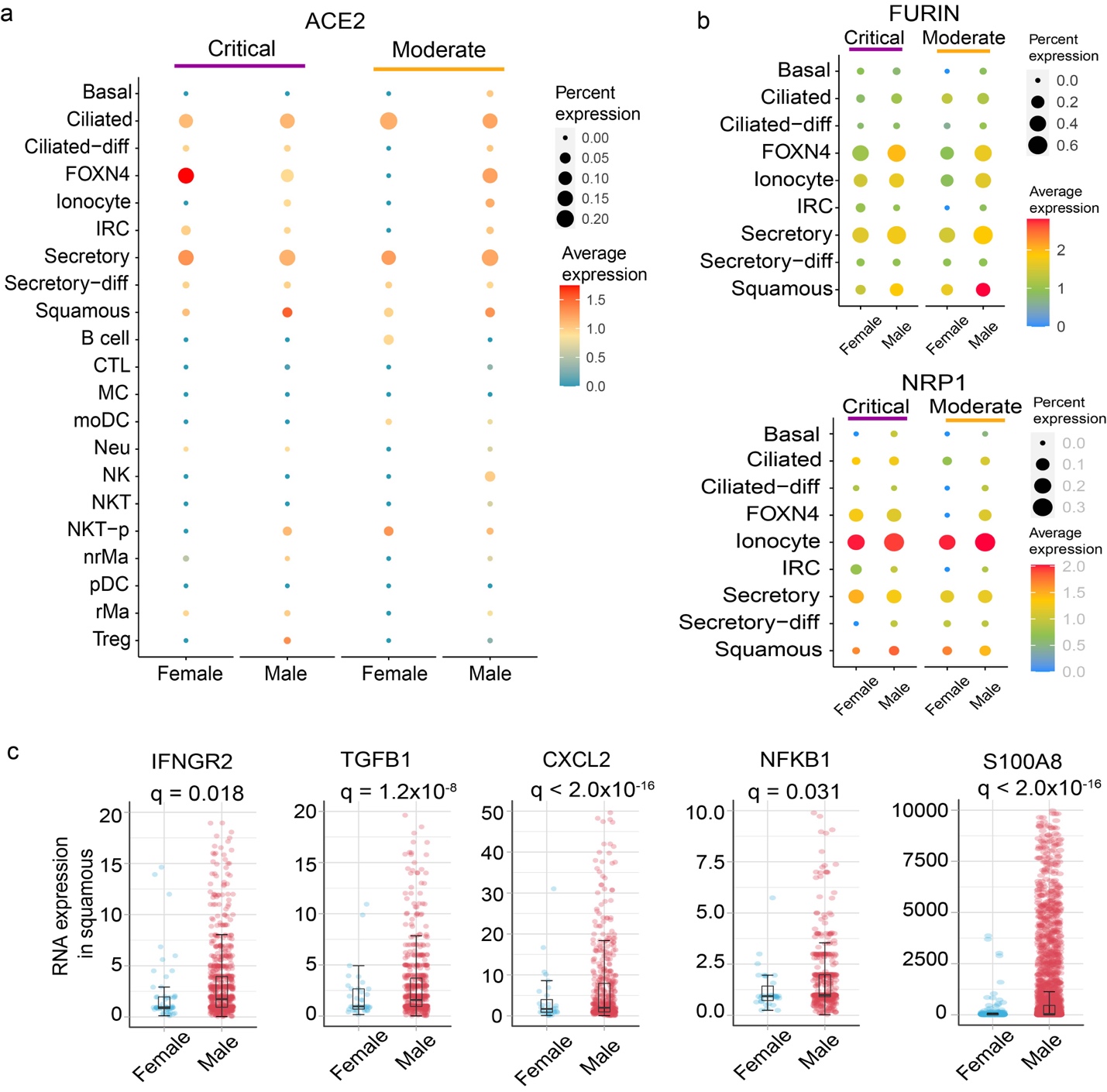
**

**Supplementary Fig. 3** **The expression of ACE2 and immune genes by sex. a** ACE2 expression by sex in 22 cell types across critical and moderate COVID-19. conditions. The size of dot denotes the percentage of ACE2 or TMPRSSE positive expressed cells. The gradient color bar represents the average expression of genes in each cell type. **b** the dot plot showed the expression level and distribution of FURIN and NRP1 in epithelial cells by sex. **c** The expression of male-biased immune genes of squamous in the patients with critical COVID-19. Each dot means one cell, and the plot only show the genes positive expressed cells. For inside boxplots, the box represents the interquartile range (IQR). Adjusted p value (q) were computed by Benjamini-Hochberg method.

**
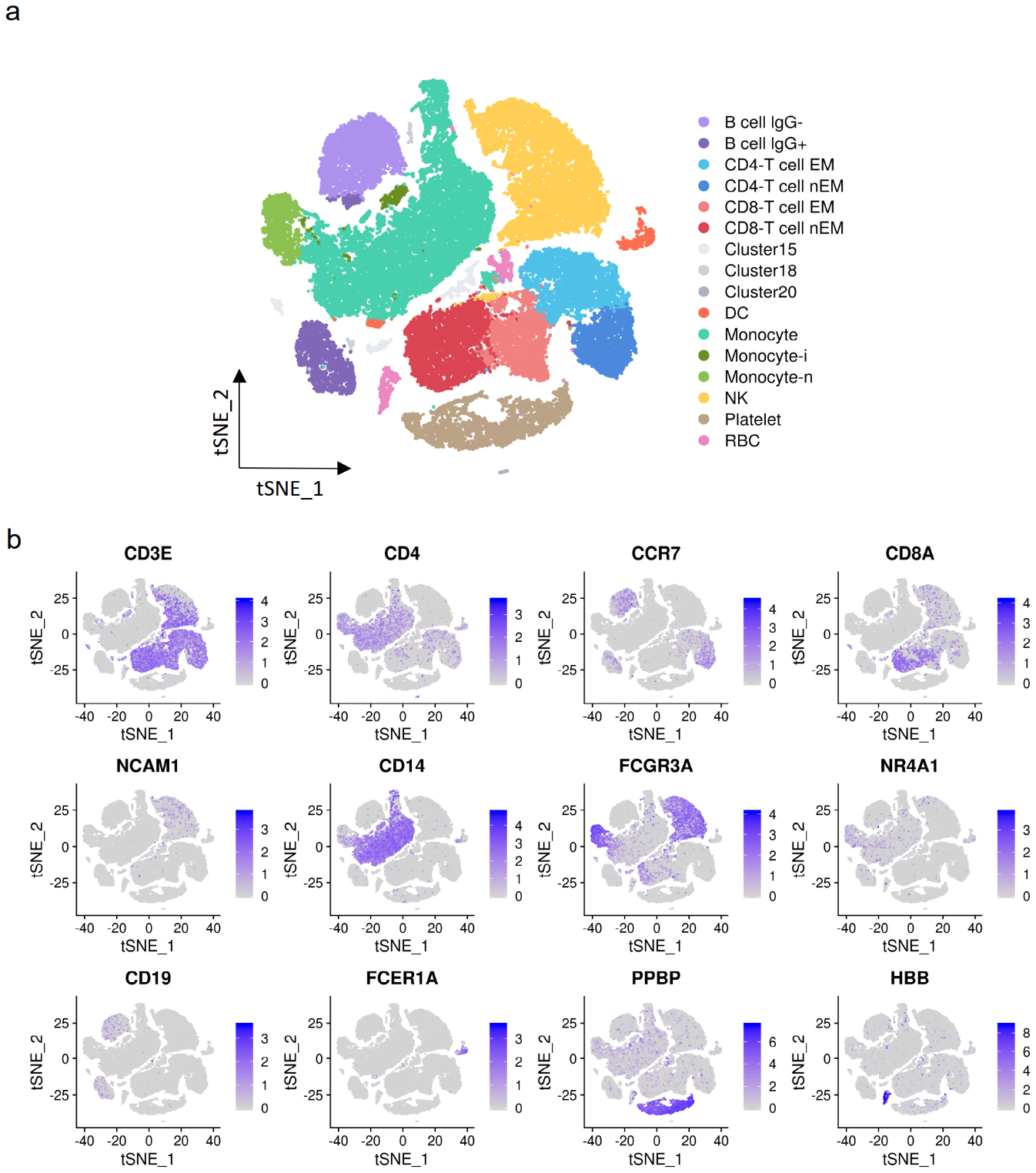
**

**Supplementary Fig. 4** **Single cell analysis of PBMC samples in COVID-19 patients and healthy donors.** **a** tSNE plot displaying all identified cell types and states. **b** The markers distribution in cell types. The expression levels are blue color coded.

**
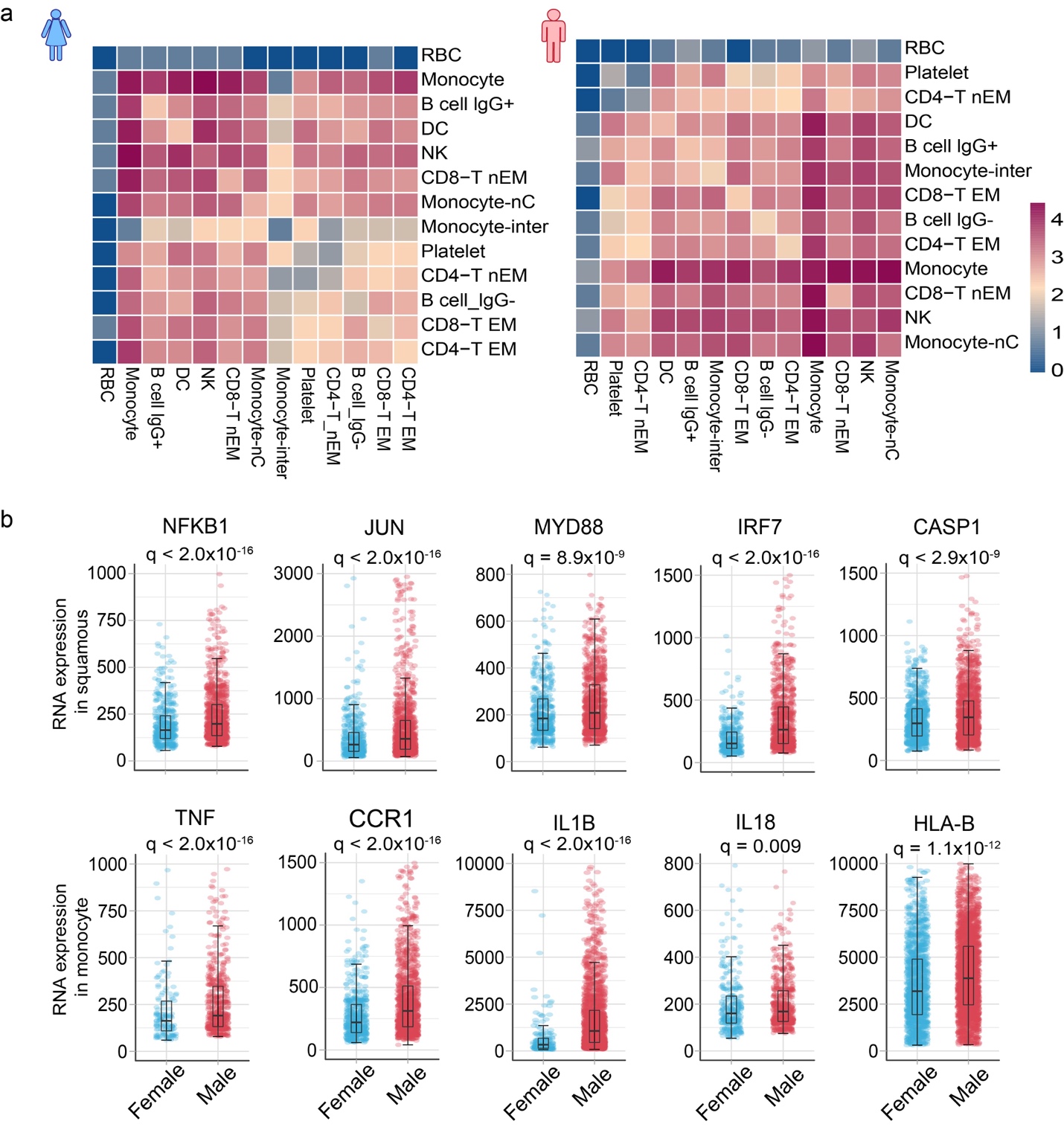
**

**Supplementary Fig. 5** **Single-cell based analysis in PBMC samples by sex.** **a** Heatmap showed the number of significant ligand-receptor interactions between cell pairs of PBMCs in male and female patients with severe COVID-19. **b** The expression of male-biased immune genes of squamous in the patients with critical COVID-19. Each dot means one cell, and the plot only show the genes positive expressed cells. For inside boxplots, the box represents the interquartile range (IQR). Adjusted p value (q) were computed by Benjamini-Hochberg method.

**
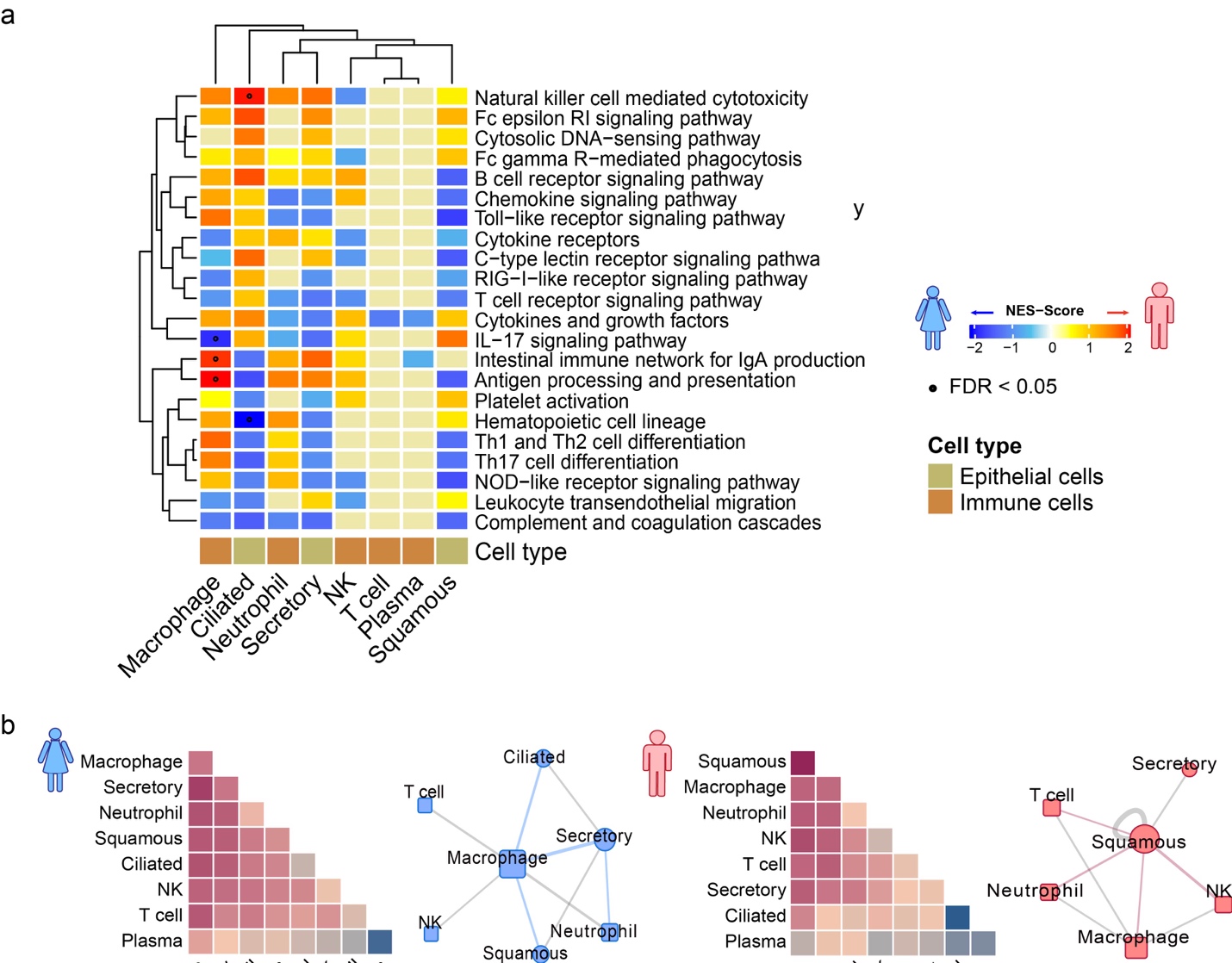
**

**Supplementary Fig. 6 Sex-biased differential cell subpopulation and transcriptional analysis for lung biospecimens. a** Gene-set enrichment analysis (GSEA) for sex-biased, differentially expressed genes across 8 cell types. The dataset includes 3,085 SARS-CoV-2 infected positive cells isolated from bronchoalveolar lavage fluid (BALF) and sputum in 6 male and 2 female severe/critical COVID-19 patients. Male-biased genes: the differentially up-regulated genes incremented by log fold changes in male patients compared with females. Female-biased genes: the differentially down-regulated genes incremented by log fold changes in male patients compared with females. The gradient color bar shows the normalized enrichment score (NES) scores. Red: the NES score > 0 and FDR < 0.05 indicate male-biased genes in a specific cell type are significantly enriched by immune pathways; Blue: NES score < 0 and FDR < 0.05 indicate female-biased genes in a specific cell type are significantly enriched by immune pathways. Black dots denote the FDR < 0.05. **b** Sex-biased cell-cell interaction network. The heatmap showing the logarithm number of significant ligand-receptor interactions between cell pairs. Number of significant interactions bigger than 50 (top 10%) was used as a cutoff to generate the cell-cell interaction network. Circle represents epithelial cell type and square represents immune cell type. The size of nodes denotes the degree (number of connections to other nodes). The colored edges represent the epithelial-immune cell connection. Other inter-connections between myeloid cells or lymphoid cells are in gray.

**
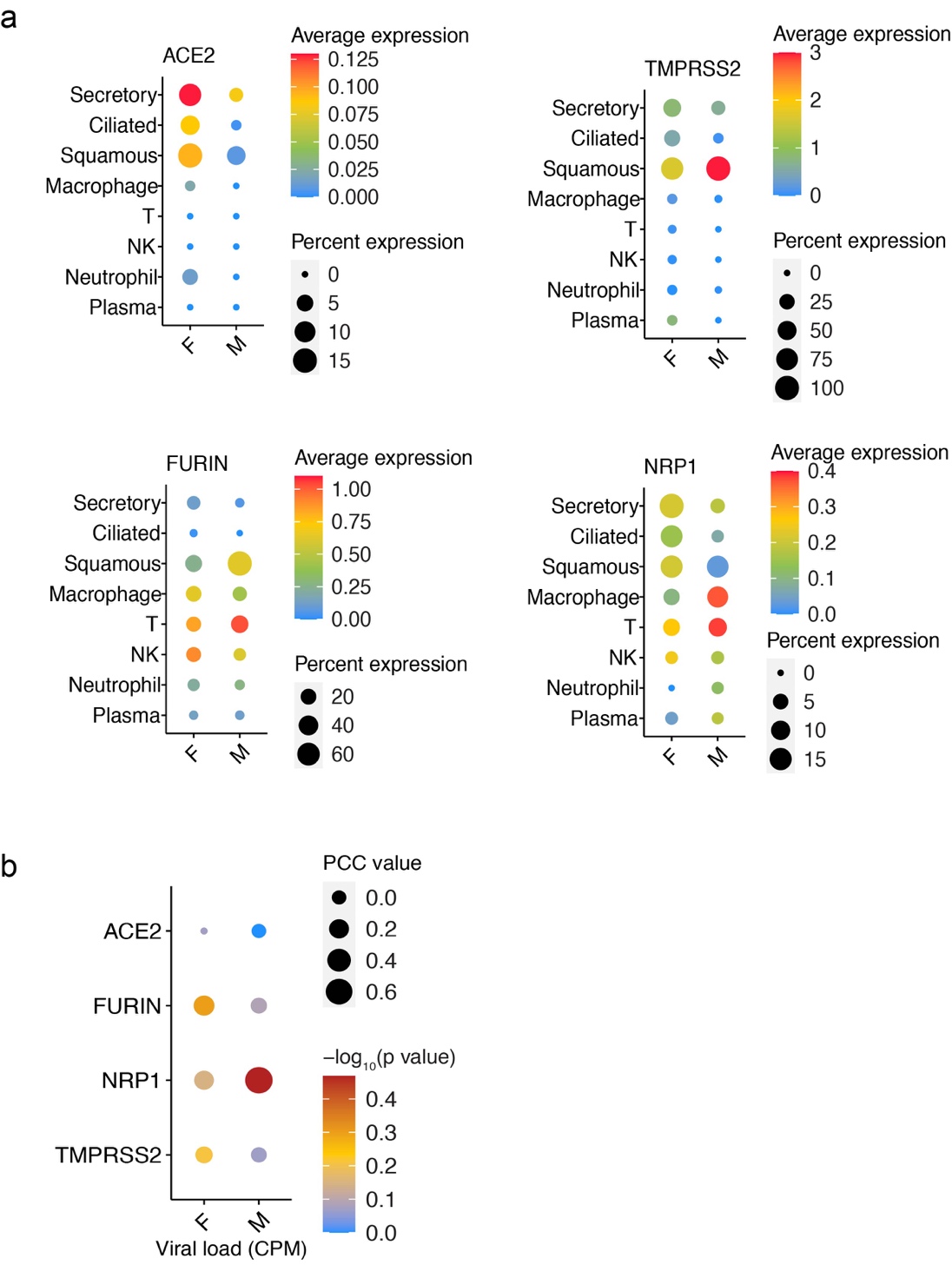
**

**Supplementary Fig. 7 SARS-CoV-2 entry genes expression analysis in lung biospecimens. a** SARS-CoV-2 entry gene expression in epithelial and immune cell types by sex. The size of dot denotes the percentage of gene positively expressed cells. The gradient color bar represents the average expression of genes in each cell type. **b** Co-relation analysis of SARS-CoV-2 viral load (reads per million) with the expression level of (reads per million) of *ACE2, TMPRSS2, FURIN,* and *NRP1*. The size of dot denotes the Pearson Correlation Coefficient (PPC) values. The gradient color bar represents the p value (F-statistics) of PCC.

**
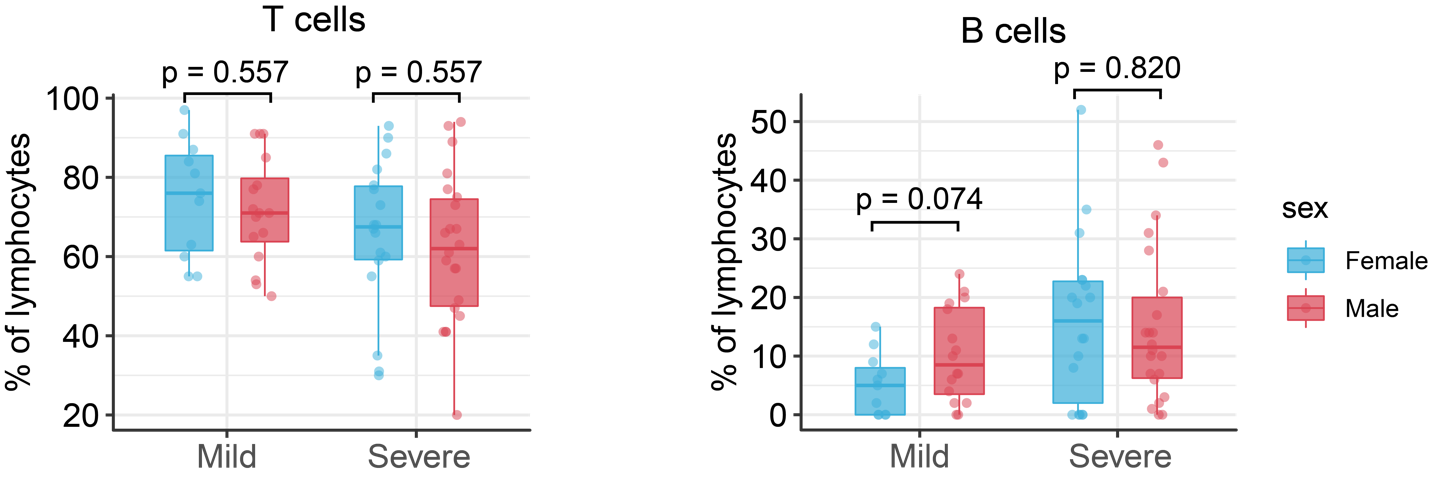
**

**Supplementary Fig. 8 Percentage of T cells and B cells in lymphocytes between female and male COVID-19 patients across two different disease severities**. In mild COVID-19 patients, we compared 16 males vs. 11 females; In severe COVID-19 patients, we compared 22 males with 18 females. p < 0.05 as significance between two groups using Wilcoxon rank-sum test.

**Supplementary Tables 1-10.**

**Supplementary Table 1**. Statistics analysis of four COVID-19 outcomes across different age groups. (.xlsx).

**Supplementary Table 2.** The patient’s information and data sources of two single-cell RNA-sequencing datasets. (.xlsx).

**Supplementary Table 3** The marker gene lists for 22 cell types of nasal samples. (.xlsx).

**Supplementary Table 4.** Summary of gene-set enrichment analysis results of a nasal tissue-based single-cell RNA-sequencing dataset. (.xlsx)

**Supplementary Table 5.** The network topological characteristics of cell-cell interaction network derived from single-cell RNA-sequencing data of nasal samples between healthy donors and COVID-19 patients. (.xlsx).

**Supplementary Table 6** The network topological characteristics of each cell type from single-cell RNA-sequencing data of PBMCs between healthy donors and COVID-19 patients. (.xlsx).

**Supplementary Table 7.** The statistical data of SARS-CoV-2 entry genes across different cell types between male and female individuals. (.xlsx).

**Supplementary Table** 8 Summary of gene-set enrichment analysis results of a PBMC-based single-cell RNA-sequencing dataset. (.xlsx)

**Supplementary Table 9** The network topological characteristics of cell-cell interaction network derived from single-cell RNA-sequencing data of PBMCs between healthy donors and COVID-19 patients. (.xlsx).

**Supplementary Table 10** The network topological characteristics of each cell type from single-cell RNA-sequencing data of PBMCs between healthy donors and COVID-19 patients. (.xlsx).
